# Transfer of conflict and cooperation from experienced games to new games: a connectionist model of learning

**DOI:** 10.3389/fnins.2015.00102

**Published:** 2015-03-31

**Authors:** Leonidas Spiliopoulos

**Affiliations:** Center for Adaptive Rationality, Max Planck Institute for Human DevelopmentBerlin, Germany

**Keywords:** transfer of learning, game theory, cooperation and conflict, connectionist modeling, neural networks and behavior, agent-based modeling

## Abstract

The question of whether, and if so how, learning can be transfered from previously experienced games to novel games has recently attracted the attention of the experimental game theory literature. Existing research presumes that learning operates over actions, beliefs or decision rules. This study instead uses a connectionist approach that learns a direct mapping from game payoffs to a probability distribution over own actions. Learning is operationalized as a backpropagation rule that adjusts the weights of feedforward neural networks in the direction of increasing the probability of an agent playing a myopic best response to the last game played. One advantage of this approach is that it expands the scope of the model to any possible *n* × *n* normal-form game allowing for a comprehensive model of transfer of learning. Agents are exposed to games drawn from one of seven classes of games with significantly different strategic characteristics and then forced to play games from previously unseen classes. I find significant transfer of learning, i.e., behavior that is path-dependent, or conditional on the previously seen games. Cooperation is more pronounced in new games when agents are previously exposed to games where the incentive to cooperate is stronger than the incentive to compete, i.e., when individual incentives are aligned. Prior exposure to Prisoner's dilemma, zero-sum and discoordination games led to a significant decrease in realized payoffs for all the game classes under investigation. A distinction is made between superficial and deep transfer of learning both—the former is driven by superficial payoff similarities between games, the latter by differences in the incentive structures or strategic implications of the games. I examine whether agents learn to play the Nash equilibria of games, how they select amongst multiple equilibria, and whether they transfer Nash equilibrium behavior to unseen games. Sufficient exposure to a strategically heterogeneous set of games is found to be a necessary condition for deep learning (and transfer) across game classes. Paradoxically, superficial transfer of learning is shown to lead to better outcomes than deep transfer for a wide range of game classes. The simulation results corroborate important experimental findings with human subjects, and make several novel predictions that can be tested experimentally.

## 1. Introduction

This study examines how dynamic, supervised learning processes operating on neural networks induce transfer of learning (ToL) from previously played games to new games. Specifically, does behavior in a new class of games (i.e., with different strategic characteristics) depend on the game class that the player was previously exposed to? And, if so, do we observe any regularities that allow us to predict the behavior of players? Path-dependence and the history of observed games can have important implications at both the micro-level (individuals) and also the macro-level (groups of individuals). At the micro-level, an individual's developmental trajectory may be seriously affected by the characteristics of the environment, such as the inherent incentives of the type of games and players that s/he is exposed to early on in development. One of the most important questions in the developmental psychology literature is how adaptation to a harsh (or safe) environment at a young age subsequently affects behavior at an older age when confronted with a different environment (e.g., see Frankenhuis and Del Giudice, 2012). For example, a nurturing school environment may be add odds with a harsher environment that adolescents face upon entering the workforce. A problematic parent-child relationship may have long-term behavioral implications even when this link is broken. At the macro-level, the aggregation of individuals' development through interactions with one another can shape the evolution of societies' functioning and culture through the endogenous emergence of expectations, social norms and conventions (Bednar and Page, 2007). For example, an initially competitive corporate environment may trap employees in sub-optimal behavior even after the environment's incentive structure is changed to foster more collaboration. On a larger scale, a country with poor rule of law and property rights may remain stuck at a sub-optimal outcome even after improving institutions. Furthering our understanding of ToL in strategic interactions is an important step in answering issues arising both at the individual and the collective level. This knowledge is relevant to numerous disciplines such as cognitive (developmental) psychology, economics, sociology, and machine learning/artificial intelligence.

Transfer of learning–also referred to as inductive transfer or knowledge transfer–has a long history in cognitive psychology dating back to Thorndike and Woodworth (1901) and plays a central role in connectionism (Pan and Yang, 2010), including the connectionist approach to cognition—see Pratt and Jennings (1996); Thrun and Pratt (2012) for extensive discussions. Despite a large literature on connectionist modeling (and ToL) for non-strategic tasks, Elman (2005, p. 113) points out that “little modeling has been done in the realm of social cognition (there is some work that looks at social interactions, but this tends to have an evolutionary focus, rather than developmental).” This study contributes exactly to this under-developed literature at the intersection of social interactions (from the viewpoint of game theory) and connectionist behavioral models.

I address the distinction between two types of qualitatively different ToL from a connectionist perspective. *Superficial ToL* manifests as behavior that is influenced by superficial similarities between different games. *Deep ToL* manifests as behavior that is influenced by structural or strategic similarities between games. The latter requires that connectionist models of strategic behavior are capable of learning higher-order (deep) representations/concepts in the first place. In the context of games, I consider higher-order representations as the strategic characteristics of games (or the incentive structure of games) in contrast to simpler representations based on the superficial similarity of payoffs across games. This raises important questions that I attempt to answer. Is ToL predominantly of the deep or superficial kind and what drives the relative prevalence of each type? Do there exist organizing principles that allow us to predict how agents will behave in a new environment, or class of games? Does deep ToL lead to higher payoffs than superficial ToL in new game classes?

The learnability (and by extension the transfer) of deep concepts has been at the forefront of the resurgence of connectionist models after the discovery of the backpropagation algorithm^1^ (e.g., Hinton, 1989; Rumelhart and Todd, 1993; McClelland, 1994). To the best of my knowledge this has not been explored systematically for strategic games with the exception of Sgroi and Zizzo (2007, 2009). These two papers explored the learnability of the Nash equilibrium concept by feedforward neural networks. However, these networks did not interact and concurrently learn from one another, but rather learned from an external teacher that provided the “correct” response (defined as the Nash equilibrium). By contrast, I examine the dynamic learning of neural networks without an external teacher, whose goal is to maximize their payoffs given the behavior of their opponents in the population. This is based on prior work by Spiliopoulos (2008, 2011b, 2012), which the current work extends to the question of ToL.

This work complements recent research in the experimental economics literature investigating ToL in games (discussed extensively in the next section). However, it is different with respect to the methodological approach and the behavioral modeling of learning. I employ a simulation (agent-based) approach that allows for the endogenous emergence of behavior arising from agent interactions. Moreover, I propose a connectionist approach to modeling behavioral learning and ToL across games with different strategic characteristics. I show how connectionist models predict and extend the robust experimental finding that prior experience in coordination games increases the likelihood of subsequent cooperation in new games of a competitive nature (Knez and Camerer, 2000; Ahn et al., 2001; Devetag, 2005; Bednar et al., 2012; Cason et al., 2012; Cason and Gangadharan, 2013; Juvina et al., 2013). Prior exposure to games with significant conflict between players–such as zero-sum, Prisoner's dilemma and discoordination games–led to significantly lower payoffs in *all* the types of games investigated in this study. The converse also holds—prior exposure to games that promote cooperation rather than conflict was more likely to lead to better payoff performance for a wide range of game classes. Furthermore, the connectionist model advances the literature by explaining how transfer of learning occurs at a computational level rather than merely describing it, and makes new testable predictions.

### 1.1. Why an agent-based approach?

Prior work in this field has tackled this problem using two approaches, theoretical and experimental. This study proposes agent-based simulations as a third methodological tool to overcome some of the limitations of the existing approaches^2^. Agent-based computational economics emphasize how the interaction of agents shapes the emergent behavior of the population—(see Tesfatsion, 2002; Tesfatsion and Judd, 2006) for an introduction and discussion of applications, and Chen (2012) for a historical overview. Schlesinger and Parisi (2001) argue in favor of agent-based computational models of cognitive development as a means of capturing the complex interaction between agents and the environment. Experiments with human subjects limit the scope of investigation due to practical constraints such as the number of subjects, treatments, amount of experience (measured by the number of games an agent is exposed to) and experimental monetary costs. By contrast, simulations are only constrained by computational costs, which are already less restrictive and subject to a decreasing trend over time. For example, running an experimental study of the analog to the simulations in this paper would require 64 different treatments, each with a large number of subjects. Furthermore, simulations can be used to initially explore a large space of possibilities and, based on the results, generate new hypotheses that can be further tested in the laboratory with human subjects.

### 1.2. Why connectionist models?

The existing experimental economics literature invokes different types of learning models driven by internal processes and agents' cumulative experience^3^. Learning over actions (e.g., reinforcement learning Roth and Erev, 1995) or beliefs (e.g., fictitious play beliefs Cheung and Friedman, 1997) severely limits models to either the same (or strategically similar) games since actions and beliefs are not invariant to the game structure. Rule-learning (Haruvy and Stahl, 2012) is more flexible, but it is still constrained by the need for the same rule to be applicable to all types of games and requires a priori specification of these rules^4^. The connectionist approach that I present can be viewed as a reductionist implementation of the rule-learning in Haruvy and Stahl (2012), since it models learning at a lower level of representation and permits the endogenous emergence of rules—see Spiliopoulos (2011b) for an example of the emergence of strategic heuristics in neural networks playing games. In the terminology of Marr (1982), rule-learning is an analysis at the algorithmic level while the connectionist models herein are closer to the implementational level. Importantly, this connectionist approach can be used to model behavior for any *n* × *n* normal form game, thereby extending the scope of learning models of strategic behavior. This permits a more thorough and broader investigation of ToL that I take advantage of by modeling behavioral spillovers across seven classes of games with different incentive structures. Another strength of this approach is that it allows the direct modeling of the emergent properties of such a learning system, especially when embedded in an agent-based framework. Munakata and McClelland (2003) advocate modeling cognitive development using a connectionist framework exactly for this reason. Mareschal and Thomas (2007) survey computational modeling in developmental psychology and contend that a computational approach is an essential step in moving from mere descriptions of behavior to explanations of behavior that provide falsifiable predictions. Connectionist models satisfy this requirement and also impart some biological plausibility to learning models by underpinning their mechanisms at the neural substrate level—see Section 2.2 for a more detailed discussion.

### 1.3. Overview

The paper is organized as follows. A literature review of both ToL studies and the implementation of neural networks to model strategic decision making follow in Sections 2.1 and 2.2 respectively. Section 3 demonstrates the detailed methods of the agent-based simulations. Section 4 presents the results and Section 5 concludes with a general discussion. Readers not familiar with feedforward neural networks and the backpropagation algorithm will benefit from a prior reading of Appendices A,B. Throughout the paper I contrast the predicted behavior from the simulations with the results from existing experimental studies conducted in the laboratory with human subjects. Also, the simulations make several novel predictions about ToL under situations that have not yet been studied in the lab.

## 2. Literature review

### 2.1. Transfer of learning

Despite the importance of ToL, the experimental game theory literature has until recently largely ignored this problem^5^. Early theoretical work, (e.g., Gilboa and Schmeidler, 1995; Samuelson, 2001; Jehiel, 2005; Steiner and Stewart, 2008), laid the groundwork for experimental investigations—the latter typically follow two learning paradigms. The *simultaneous* learning paradigm exposes subjects to a set of strategically different games and contrasts this with treatments where subjects were exposed only to a single class of games. The *sequential* learning paradigm repeatedly exposes subjects to the same game (or perhaps different games belonging to the same class). Empirical evidence from these paradigms finds that agents transfer learning from one game to another. However, the degree of ToL is mediated by other variables, such as the complexity of the game and opponents' behavior, the degree and type of similarity (deep or superficial) between games.

As mentioned in the introduction, ToL can be driven from two different sources. Knez and Camerer (2000) differentiate between ToL arising from *descriptive* and *payoff* similarities of the new game compared to previously experienced games. Descriptive similarity refers to the action (choice) labels, the number and identity of players, and the presentation format of a game. Payoff similarity refers to the strategic characteristics of a game, which ultimately are a function of the payoff and action spaces of all players. They present experimental evidence that ToL between Prisoners' dilemma and Weak-link games depended strongly on the descriptive similarity of the games. Juvina et al. (2014) also differentiate between ToL arising from descriptive and payoff similarities; note, they refer to them as *surface* and *deep* similarities respectively. They conclude that both types of similarity are important, and ToL was strongest when both surface and deep similarities suggested the *same* behavior in the new game. Another important result is that deep transfer can occur even when surface similarity is absent; indeed, the existence of surface similarity can hinder transfer based on deep similarity. For example, Rick and Weber (2010) find that subjects learn the notion of iterated dominance and transfer its use to similar but new games; however, such deep transfer was more prevalent when feedback was suppressed. Haruvy and Stahl (2012) find significant evidence of deep ToL, and an increase in the depth of reasoning in dissimilar 4 × 4 normal form games.

Cooper and Kagel (2003, 2007) document the importance of sophisticated learners in facilitating the transfer of knowledge in limit-pricing and signaling games. Subjects repeatedly playing two different games against two fixed opponents in Bednar et al. (2012) exhibited both behavioral spillover (using similar strategies across games) and non-optimal play due to cognitive load. Devetag (2005) finds that a precedent of efficient coordination in the critical-mass game carries over to play in a minimum-effort game. Cason et al. (2012) find significant spillovers when subjects sequentially played a median-effort coordination game followed by a minimum-effort coordination game. The Pareto-optimal equilibrium in the minimum-effort game was more likely when players coordinated in the previously played median-effort game. Mengel and Sciubba (2014) conclude that prior experience with a structurally similar game leads to faster convergence to a Nash equilibrium (NE). Conversely, prior experience with a structurally different game leads to less coordination and a lower probability of Nash equilibrium play.

In this paper's setting, learning takes place in a more demanding environment than the majority of experimental studies. Agents are required to learn to play randomly generated games drawn from a single *class* of games, not a single game that is repeated before testing transfer to a new game. The closest experimental paper using a similar setup is Grimm and Mengel (2012) in which games were randomly drawn from a set of two or six different classes and players were randomly rematched after every round.

### 2.2. Neural network models of strategic learning

Studies modeling decision makers as neural networks are relatively scarce, but increasingly attracting more attention. Sgroi and Zizzo (2007, 2009) find that neural networks can learn to use heuristics approximating the Nash equilibria in 3 × 3 normal form games when receiving feedback from a teacher. Spiliopoulos (2008, 2011b, 2012) extend this research to tabula rasa neural networks *concurrently* learning to play 2 × 2 or 3 × 3 normal form games, without an external teacher to provide the “correct” response. Regret-driven neural networks can predict subjects' behavior in games with a unique mixed strategy Nash equilibrium, both when repeatedly playing a single game (Marchiori and Warglien, 2008), and when concurrently learning to play different instances of such games (Marchiori and Warglien, 2011). Note, their setup used instances of games drawn *within* the same game class, not *across* game classes as I propose. Similarly to human subjects, NNs learning to play two-stage games with a unique subgame-perfect Nash equilibrium exhibit bounded rationality (Spiliopoulos, 2011a); specifically, subgame and truncation inconsistency.

This paper extends the methodology of Spiliopoulos (2008, 2011b, 2012) to investigate ToL across different game classes—a summary of the main results and advantages of using this methodology follows. The NN agents in these studies were randomly matched and played randomly chosen 2 × 2 and 3 × 3 normal form games regardless of their strategic characteristics such as number and types of equilibria. The ability of these NNs to produce a valid response for *any n* × *n* game–regardless of whether it has been observed before or not– makes them a viable model of ToL. The main results in Spiliopoulos (2012) for 2 × 2 games are: (a) NN agents learned to play the pure-strategy Nash equilibrium of different classes of games with near certainty, and (b) NNs learned to adhere to principles of dominance and iterated dominance with near certainty. The main results in Spiliopoulos (2011b) for 3 × 3 games are: (a) NN agents learned to behave similarly to human subjects in the lab with respect to a number of criteria, such as employing similar heuristics, equilibrium selection, use of the principles of dominance and iterated dominance, and (b) the endogenous emergence of a similarity measure of games based on the number and type of Nash equilibria.

The use of neural networks has numerous advantages—the reader is referred to Spiliopoulos (2008, 2011b, 2012) for extensive arguments. Similarly to the human brain, NN agents encode knowledge in a parallel-distributed topology and learn using a simple rule, the backpropagation (BP) algorithm (Rumelhart et al., 1986) that is driven by ex post best-response. The BP algorithm is simple and effective, requiring only first-order gradient descent calculations ignoring second-order information. Originally, the biological plausibility of the BP algorithm was not taken literally, as it required a global teacher and evidence of its existence in the human brain was lacking. This view is changing as evidence is accumulating that neuromodulators, such as dopamine, may provide the global learning signal required for supervised learning, (e.g., Egelman et al., 1998; Schultz, 1998; Glimcher, 2011). See Zipser and Andersen (1988); Mazzoni et al. (1991) for arguments that the backpropagation algorithm reflects the same algorithm used in the brain, and Robinson (2000); van Ooyen and Roelfsema (2003) for arguments that BP may be approximately equivalent (or easily modified) into an algorithm that is biologically plausible^6^.

## 3. Methods

### 3.1. Game classes

This study uses seven different classes of 2 × 2 games from the literature, chosen according to the requirements that they be widely-studied games with a diverse range of strategic characteristics. Table 1 lists the game classes and compares the following characteristics (for generic games)^7^: #PSNE (the number of pure strategy NE), #MSNE (the number of mixed strategy NE), #PDNE (payoff-dominant NE), #RDNE (risk-dominant NE), and whether a game is dominance solvable. These game classes include diverse social interactions where players may be in direct competition (e.g., ZS), no competition or no conflict (NC), and games where both cooperation and conflict coexist. Coordination games have multiple equilibria and provide incentives for players to coordinate on the same actions. Theories of equilibrium selection seek to explain which equilibrium is more likely to be attained (Harsanyi and Selten, 1988). Anti-coordination games provide incentives for players to settle on different actions. Discoordination games exhibit a mixture of both coordination incentives (for one player) and anti-coordination incentives (for the other player). Such games allow only for a unique mixed strategy NE. Games of pure conflict do not incentivize any kind of cooperation as one player's gain is necessarily another player's loss. Social dilemmas, such as Prisoner's dilemma, have elements of both conflict and cooperation leading to a Nash equilibrium that is sub-optimal for both players. Detailed taxonomies of 2 × 2 games can be found in Rapoport et al. (1976); Kilgour and Fraser (1988).

**Table 1:** **Game class characteristics**.

**Game class**	**Abbreviation**	**Number and types of Nash Equilibria**			**Dom. solvable?**	**Incentives**
		**PSNE [PDNE, RDNE]**		**MSNE**		
Zero-sum	ZS	0 or 1		1 or 0	Possibly	Pure conflict
Prisoner's Dilemma	PD	1		0	Yes	Social dilemma
Mixed strategy	MS	0		1	No	Discoordination
Stag hunt	SH	2	[1, 1]	1	No	Coordination
Chicken	CH	2	[0, 1]	1	No	Anti-coordination
Battle of the Sexes	BOS	2	[0, 1]	1	No	Coordination
No competition	NC	1		0	Possibly	No conflict

PSNE, pure strategy NE; MSNE, mixed-strategy NE; PDNE, payoff-dominant NE; RDNE, risk-dominant NE.

### 3.2. Neural network agents

Each agent is modeled as a feedforward neural network consisting of an input layer, three hidden layers and an output layer—see Appendix A for more details. The input layer consists of eight neurons, each of which receives an input from one of the eight payoffs of the 2 × 2 normal form games. Each hidden layer consists of fifty neurons, which perform *tansig* transformations. The output layer consists of two neurons, one for each of an agent's possible actions. Their output can be interpreted as a probability distribution over a NN's action space as they perform a softmax (or logit) transformation (Spiliopoulos, 2008, 2011b; Marchiori and Warglien, 2011). Finally, a stochastic decision rule randomly determines the realized action. The correct response for an agent is determined by the principle of ex-post rationality (Selten, 1998)—this has also been previously used to model NN learning (Marchiori and Warglien, 2008; Spiliopoulos, 2008, 2011b). An agent computes the correct response (after observing the outcome of the round), defined as the myopic best response to the opponent's action. After every game, a standard online back-propagation algorithm adjusts the NNs' weights in the direction of the ex-post best response—see Appendix B for more details.

This paper focuses on the effects of nurture or the endogenous emergence of preferences (risk and social) whilst making minimal assumptions regarding nature or exogenously imposed preferences—see Zizzo (2003) for a discussion of nature vs. nurture and endogenous vs. exogenous preferences in the context of economic decision making^8^. Nurture operates through the exposure to specific game classes and opponents' behavior. Of course, some assumptions must be made regarding nature–I have strived to keep these as minimal or broad as possible. By nature, I refer to the chosen architecture of the NNs, the characteristics of the neurons' transfer function and the learning mechanism (backpropagation). The choice of number of neurons and hidden layers was made on the basis of prior research showing that this level of complexity was both necessary and sufficient for NNs to approximate observed human behavior in experiments (Spiliopoulos, 2011b, 2012). The backpropagation algorithm discussed above was deliberately chosen for its relatively agnostic view on learning that imposes virtually no specifications on an agents' utility function. The only assumption in the computation of the specified backpropagation algorithm is that an agent prefers a larger payoff to a smaller payoff. In contrast to other possible implementations of the learning algorithm, the magnitude of the difference in payoffs between best-responding and not best-responding is irrelevant. For example, the size of weight adjustment could be directly linked to the magnitude of regret; however, this would require the specification of a cardinal utility function and an assumption regarding risk preferences.

Despite the risk-neutrality of the backpropagation algorithm, the use of a tansig transfer function for the non-output layer neurons implicitly embeds some risk-aversion into system. The use of bounded transfer functions is unavoidable as high input values would otherwise propagate through the network leading to instability. Real neurons also exhibit saturation in their firing rates with increasingly large inputs, therefore it is desirable to use a similar function in simulated neurons. Furthermore, consider that the learning algorithm deals solely in an agents' *own* payoffs. Consequently, agents' risk and social preferences–that can be inferred from learned behavior–should be considered endogenous (or constructed) rather than exogenous.

Finally, note that if NN agents learn to always play a PSNE then the whole system is at a steady-state. Since each player is choosing a best response to the opponent, the error of all networks is equal to zero; therefore, no adjustment is made to any NN weights^9^.

### 3.3. Simulation details

The set of simulations are divided into eight training sessions and eight testing sessions, for a total of sixty-four combinations. Each training and test session uses one of eight sets of games: seven sets corresponding to each of the classes defined earlier, and the eighth set, denoted as ALL, consisting of games drawn with equal probability from each of the seven classes. Each training simulation consisted of a population of ten NN agents that were presented with 70,000 randomly drawn games from the training set and were randomly rematched with an opponent for each game. Agents had perfect information about the game and received feedback about the action that their opponent played. During the training sessions the NNs learn how to play the games they are exposed to. The structure of the NNs is then fixed (i.e., learning stops), and their behavior for the test set consisting of one thousand games is simulated. This results in an 8 × 8 set of comparisons revealing the path-dependence, or relationship, between the class of games used in the training set and the subsequent behavior of the NNs on the game classes of the test sets. ToL is revealed by comparisons of how NNs trained on different training sets behave for each specific test set.

Training NNs on the ALL set parallels the *simultaneous* learning paradigm, similar to the experimental setup of Rankin et al. (2000) and to the NN learning simulations of Spiliopoulos (2008, 2011b, 2012). Training on a single class of games and subsequently testing on another game class parallels the *sequential* learning paradigm. The games for the training and test sets were sampled using the GAMUT suite of game generators (Nudelman et al., 2004). All simulations were performed in Matlab using a combination of custom code and functions from the Matlab Neural Network toolbox.

## 4. Results

The following subsections present the results obtained from the NN simulations. Since simulations allow for an arbitrarily large number of samples (in this case, the number of games used to compare behavior), inferential statistics are not informative. Consequently, I do not report *p*-values but instead focus on the effect size or economic significance of the results. Throughout the paper, hybrid table/heat-maps are used to aid interpretation and comparisons—the higher a table cell's value the darker its background shading. A number of important results are highlighted and numbered—these may be comparable to existing empirical results or may make new predictions about behavior that has not yet been investigated in the lab with real subjects. In the latter case, the number of the result is followed by an asterisk to denote that this is a novel and testable prediction, e.g., Result 2^*^.

### 4.1. Convergence of simulations

**Figure 1:**
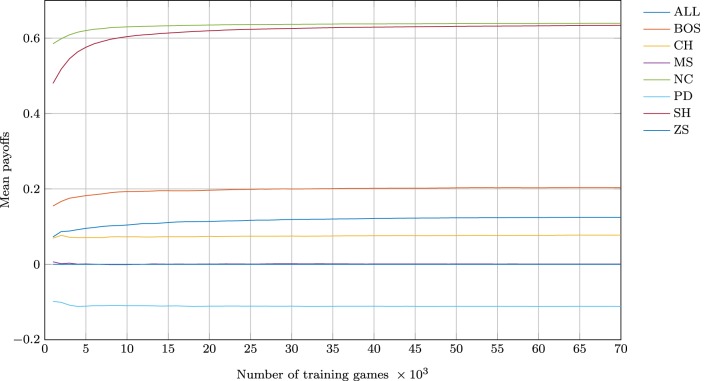
**Convergence of the simulations—mean payoffs as a function of the number of games played**.

Before proceeding with detailed analyses, I establish that all eight training simulations have converged. Figure 1 plots the mean payoffs of the NN agents against their cumulative experience, i.e., the number of training games they have been exposed to. It is clear that training the NNs for 70,000 games is more than adequate for the convergence of all simulations—most converge with as little as 10,000-20,000 presentations.

### 4.2. Agent homogeneity/heterogeneity

This section examines whether agents' emergent behavior exhibits significant heterogeneity either due to the initial randomized starting weights of the NNs or individual-specific differences in experience. The high degree of stochasticity in the learning process minimizes the possibility of strong dependence on initial values and the probability of the whole population (or subsets of it) converging on different local solutions. This stochasticity works through many channels: (a) stochastic choice as implemented by the softmax function in the output layer, (b) random rematching of players after every round, (c) randomization of the game payoffs after every round. For each of the eight training sessions, I subsequently present the NNs to one thousand games from the same class that they were trained on. The following statistics are based on the predicted choice probabilities of each NN agent for every game. For each game class, I calculate the Spearman (rank) correlation coefficient between the choice probabilities of every possible pairing of the ten NN agents. Similarly, I simulate the realized choices of each NN agent and determine the probability that each pair of agents chose the same action in each game—this is referred to as choice agreement. Table 2 reports the mean, minimum and maximum values of these statistics calculated over all possible pairs of NN agents.

**Table 2:** **Agent heterogeneity—rank correlation of choice probabilities and % choice agreement**.

	**Rank correlation**			**Choice agreement (%)**		
**Game class**	**Mean**	**Min**	**Max**	**Mean**	**Min**	**Max**
ZS	0.96	0.94	0.98	80	77	82
PD	0.83	0.67	0.93	100	99	100
MS	0.01	−0.54	0.52	50	45	59
SH	0.87	0.79	0.94	100	99	100
CH	0.55	0.19	0.90	61	57	65
BOS	0.86	0.69	0.96	87	85	90
NC	0.90	0.84	0.94	100	99	100
ALL	0.93	0.89	0.95	72	68	76

The mean correlations for each game class (or equivalently, training simulation) are very high (ranging from 0.83 to 0.96) with two exceptions—classes MS and CH, 0.01 and 0.55 respectively. The minimum and maximum correlations are similarly quite tightly concentrated around the mean values, indicating relatively homogeneous populations of agents.

The exception of the MS class is not surprising if one considers that these games do not provide a strong consistent learning signal as the NE behavior is a mixture over actions. By the definition of a MSNE, even if an opponent is playing according to the MSNE, a player has no incentive whatsoever to also play the MSNE as her expected payoffs are identical. This hinders the emergence of the MSNE in the long run, in contrast to other games where a PSNE exists; players have an incentive to play the PSNE if their opponent is playing it.

The source of the higher degree of heterogeneity discovered for the CH game becomes clear if one considers that the NN agents in the simulation are unaware of the identity of their opponent. Since CH is an anti-coordination game, a PSNE can only be consistently played by agents if they are able to identify each other. This requires the existence of an uncorrelated asymmetry that is absent in these simulations. Consequently, the only *symmetric* equilibrium of the CH game is the MSNE of the game. Simulation results presented in Section 4.5 support this as the probability of agents in the CH game playing the PSNE is significantly lower than in other games. This is true even in comparison with other game classes that also have two PSNE and one MSNE such as SH and BOS—the key difference is that CH is an anti-coordination game whereas SH and BOS are not.

Consequently, throughout the paper I discuss population-level statistics of the emergent behavior of the NN agents. Due to the high correlation in behavior within a training simulation, these can also be interpreted as individual-level characteristics, with the exception of the MS and CH simulations.

### 4.3. Actions

The hypothesis of path dependence and experience-dependent ToL can be captured by the degree of correlation of the NNs' behavior (measured by the probability distribution over actions on the games of the ALL test set) for all possible pairwise comparisons of the training game classes. These are presented in Table 3—a hypothesis that prior experience is irrelevant is consistent with all the correlation coefficients being equal^10^.

Result 1: There exists significant transfer of learning, i.e., behavior in a new test game class depends on the training game class.

**Table 3:**
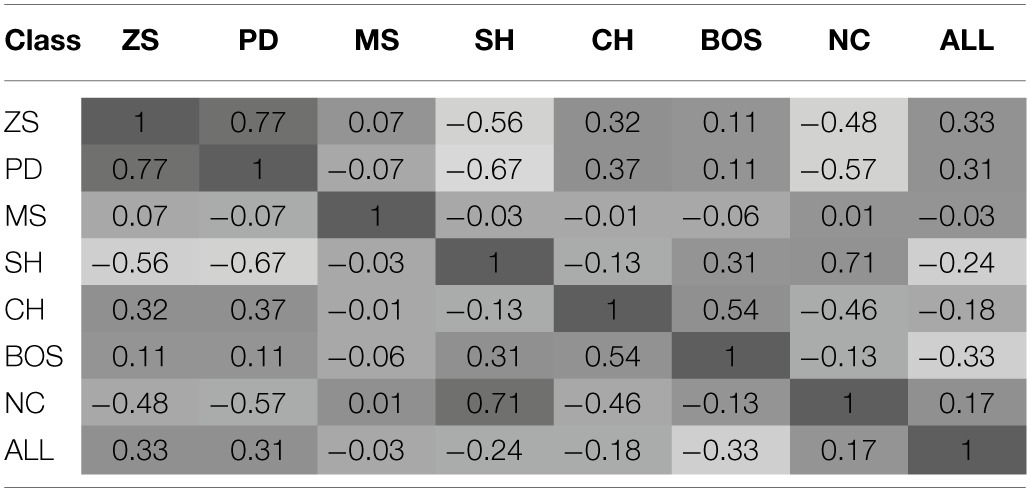
**Spearman rank correlation of agents' behavior by training sets**.

The higher a cell's value, the darker the shading.

The hypothesis of no ToL can be rejected as the correlation coefficients are highly heterogeneous, with values ranging from −0.67 to 0.77. This confirms the findings of the experimental studies discussed in Section 2.1.

Result 2^*^: The degree of similarity of NN behavior in the test sets is predicted better by the games' strategic characteristics (degree of conflict or mutual interests) than the number (and type) of Nash equilibria of the training game class.

The largest positive correlations are found for these pairs of training sets {*PD*, *ZS*} {*NC*, *SH*} {*BOS*, *CH*}, whilst the largest negative correlations are for the pairs {*SH*, *PD*} {*NC*, *PD*} {*SH*, *ZS*}. Comparing training classes based on the number and type of NE leads to the observation that learned behavior is significantly different. For example, the two game classes with a single PSNE (PD and NC) exhibited highly different behavior as exemplified by a strong negative correlation, −0.57. Game classes with two PSNE led to more similar behavior, but still far from a perfect correlation. Similarly, note that the second highest correlation occurs between NC and SH; these are game classes that differ in their number of PSNE, but share a high degree of mutual interest (i.e., the payoff-dominant NE).

Comparing training classes based on their strategic characteristics leads to more consistent behavior. Both NC and SH classes have a PSNE where all players achieve their highest possible payoff (and consequently is the socially efficient outcome), i.e., there is no conflict in these two game classes. Similarly, behavior after training on PD was most similar to that of ZS; note, that both of these games have strong elements of conflict. Concluding, NNs trained on game classes with similar strategic characteristics behave more similarly in new game classes than NNs trained on game classes with the same number and type of NE—this is indicative of ToL arising from payoff similarity or deep transfer.

### 4.4. Payoffs

**Figure 2:**
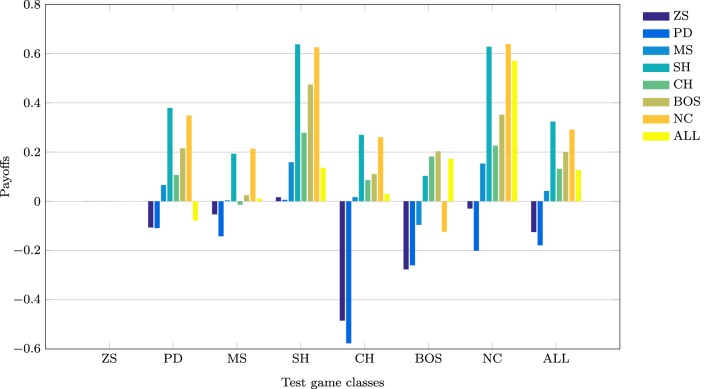
**Mean payoffs (conditional on the training and test sets)**.

This section discusses the payoff performance of NNs conditional on the pairing of training and testing sets—see Table 4 and Figure 2 for a graphical presentation. Furthermore, define a game class to be ToL dominated if there exists at least one other game class that has higher expected payoffs for *each* of the seven game classes. Table 4 also reports whether training on a game class was dominated (column Dom'ed?) and which game classes, if any, a particular game class dominates (column Dom'es).

Result 3: Maximum payoffs are achieved when players have prior experience with games where interests are aligned: SH games, closely followed by the NC games.

**Table 4:**
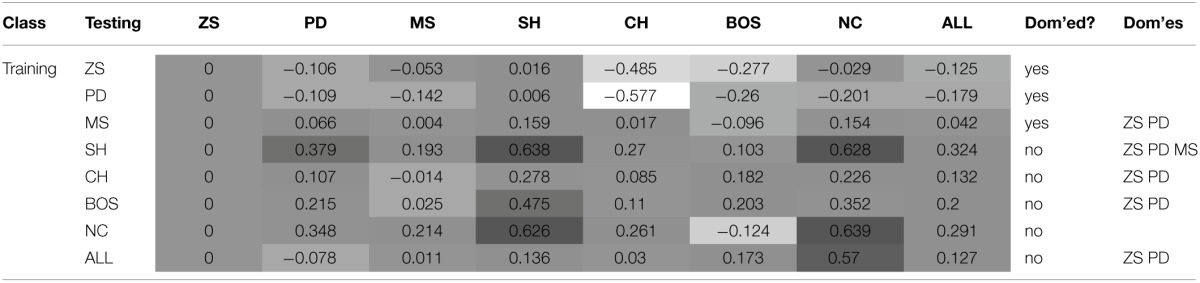
**Mean payoffs (conditional on the training and test sets)**.

The higher a cell's value, the darker the shading.

The best payoff performance is achieved by networks trained on the SH set. This is true both in terms of the highest payoff for the ALL test set (0.324) and also the number of individual game classes for which maximum performance is achieved (PD, SH, and CH). The next best performance is attributed to networks trained on the NC dataset, which achieve the highest payoffs in the MS and NC games. Note, that the interests of both players are aligned in the SH and CH classes. The following converse result corroborates this finding.

Result 4: Minimum payoffs are achieved when players have prior experience with games where conflict is prevalent: PD games, closely followed by ZS games.

The worst performing training set is the PD class, as it has the lowest payoff for the ALL set −0.179, the worst performance in five game classes, and the second worst performance in another game class. Consistent with the results in Duffy and Ochs (2009), cooperation is not found to emerge in the PD game when players are randomly-matched, as is the case with these simulations. The second worst performance occurs for zero-sum games, corroborating Result 3, as both PD and ZS game classes have strong elements of competition/conflict rather than cooperation. This is true by definition for zero-sum games where every outcome is zero-sum, whereas in the PD games the maximum outcome for one player leads to the worst outcome for the other player in two cells. This striking result has important implications as the PD game is regarded an one of the archetypal games assumed to describe many interactions in the real world. NNs trained on the PD game class learn to avoid the socially efficient (non-Nash equilibrium) outcome because of the risk associated with an opponent deviating from this outcome. Consequently, this strongly influences agents to learn the pure strategy Nash equilibrium, which in some game classes leads to socially inefficient outcomes. As discussed later, NNs trained on the PD set learn to play the NE of the PD game almost perfectly, and in SH games–where there exist two PSNE–they choose the risk-dominant NE 99% of the time. This is consistent with learning about the significant deviation costs (and risk) associated with the PD game and transferring this to the SH game.

Result 5^*^: Training on ZS, PD or MS game classes is dominated by training on at least one other game class.

This result means that regardless which of the seven game classes are to be played in the test set, it is *never* optimal to have prior experience with one of these three game classes. This result strengthens and generalizes the empirical findings regarding exposure to the PD class—in particular, we are unaware of experimental studies that use ZS or MS as the training set to test this novel prediction.

Result 6: Cooperation in the PD game can be enhanced if it is preceded by experience with coordination games.

Ahn et al. (2001) find that playing a coordination game before a PD game leads to increased cooperation in the latter, both for fixed- and random-matching of players (the effect is stronger for fixed-matching). Similarly, Knez and Camerer (2000) hypothesize that cooperation can be increased in the PD game if it is preceded by the SH and players have a history of playing efficiently. Indeed, the highest payoff in the simulations for the PD test set occurred when it was preceded by the SH game. Juvina et al. (2014) find that fixed-pairs of players are more likely to achieve the cooperative outcome in PD when they had prior experience with the CH game. This is also confirmed by the simulations as the payoffs in PD were significantly higher when NNs were trained on the CH class (0.107) than on the PD class (−0.109).

The lowest payoffs occur when PD is preceded by PD or ZS, i.e., games with significant conflict of interests. Also, as shown later, NNs trained on SH generally show a preference for the payoff-dominant NE, i.e., the socially efficient outcome. Furthermore, the second highest payoff to PD is attained when it is preceded by another game where conflict is absent, the NC class.

Result 7^*^: Transfer of learning from the NC to the BOS game class leads to relatively low payoff performance.

Despite the fact that training on the NC class of games leads to the second highest average payoff for the ALL test set, it exhibits particularly poor payoff performance–the third worst– for the BOS game. It is the poor performance only in the BOS test class that prohibits training on NC from dominating other game classes. It remains to be seen whether this prediction of relatively poor performance only for the BOS test set is verified experimentally in the lab.

### 4.5. Attainment of nash equilibria

**Figure 3:**
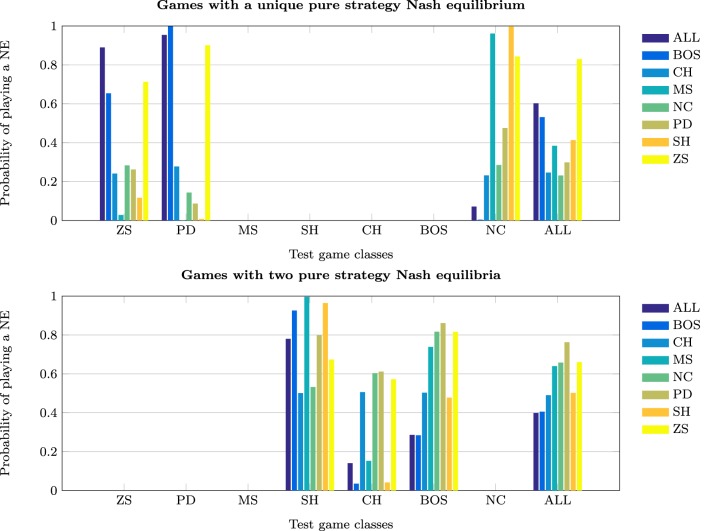
**Probability of playing Nash equilibria**.

This section investigates the effect of exposure to the training set on the probability of subjects jointly playing a PSNE in the test set—see Tables 5 and 6, and Figure 3.

*Result 8: The probability of playing a unique Nash equilibrium in the test sets is greatest when players are simultaneously trained on all game classes (rather than sequentially trained on any single game class). This is in conflict with the prediction made by the cognitive-load hypothesis* (Bednar et al., 2012).

**Table 5:**
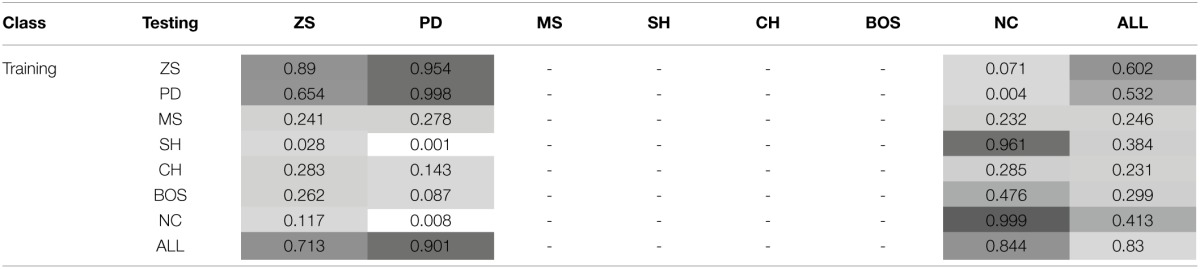
**Joint probability of playing a PSNE (conditional on the training and test sets)—Games with a unique PSNE**.

The higher a cell's value, the darker the shading.

**Table 6:**
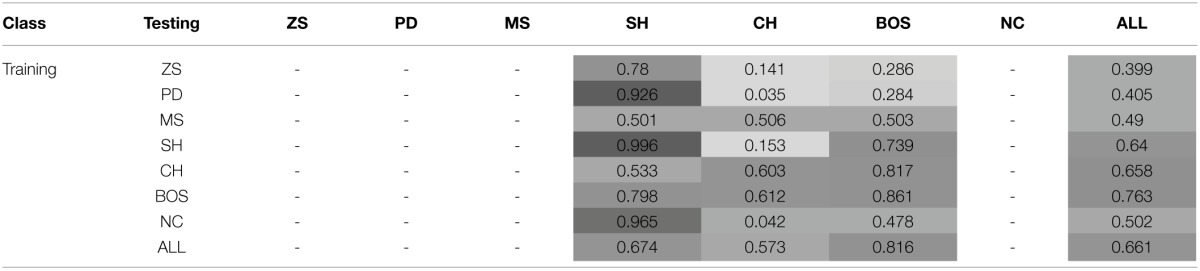
**Joint probability of playing a PSNE (conditional on the training and test sets)—Games with two PSNE**.

The higher a cell's value, the darker the shading.

The probability of joint PSNE play is maximized when the NNs are trained on the ALL test set, 0.83. The next best performance occurs when the NNs are trained on ZS and PD. However, while these perform well on ZS and PD test sets, they perform very poorly on the NC class. This is the opposite of what is observed when training on the NC class; it performs very poorly on ZS and PD but extremely well on NC. In conjunction with prior results, this strengthens the argument made that learning to play game classes with the element of conflict such as PD and ZS, is quite different from learning to play games without conflict.

The finding that training on the ALL test set is conducive to achieving a Nash equilibrium outcome in the test classes is, at first sight, surprising. The cognitive-load hypothesis (Bednar et al., 2012) states that simultaneous play of strategically different games may lead to less efficient or even non-equilibrium behavior as subjects may choose similarly. An important difference that can explain this disparity is that in experimental studies experience with games is severely limited compared to the simulations reported herein. Simultaneous learning of games may initially impair learning, but become conducive to learning the NE over time as experience accumulates. An alternative explanation for this finding is that training on a single game class increases the likelihood that the NNs will learn a simple heuristic that may guarantee Nash behavior only in that specific class. Thus, training on a limited set of games may encourage superficial learning and impair deep learning. For example, in the NC class the NE can always be achieved by each player choosing his/her action with the maximum payoff (or the action that is socially efficient). Such a heuristic would be effective in the SH class, as it would lead to a preference for the PDNE over the RDNE. Indeed, NNs trained on NC games perform exceptionally well in SH games. However, such a heuristic would not be as effective in BOS as each players' maximum payoff corresponds to different actions. Therefore, there is the strong possibility of discoordination arising, which is actually observed in the simulations. Note also, the similarity in the results for populations trained on the SH and NC classes. In both cases, the maximum payoff heuristic would lead to a NE, and in the case of SH to the Pareto-optimal NE.

In test classes with two PSNE, the highest probability of jointly playing one of the PSNE is achieved by training on the BOS game classes (0.763), followed by training all the ALL test set. Highly competitive training game classes, such as ZS and PD, perform poorly, i.e., below the chance rate of 0.5 for random play.

### 4.6. Equilibrium selection

In this section, I examine how prior learning or exposure to specific game classes can subsequently affect equilibrium selection. Note that no risk preferences were embedded by design into the NN agents, therefore the preferences over the types of equilibria occur solely through agents' exposure to and learning of other games. The stag-hunt game class is of particular interest, as it has distinct payoff- and risk-dominant equilibria. Equilibrium selection between these two types of equilibria has been of ongoing interest in the literature. Numerous experimental studies find that risk-dominant NE tend to be played more often than payoff-dominant NE (Straub, 1996; Cabrales et al., 2000), especially as payoffs become more asymmetric. However, Schmidt et al. (2003) find that payoff-dominant NE are observed more frequently, although subjects' behavior was mediated by risk-dominance properties. Also, Rankin et al. (2000) use a series of perturbed games (instead of identical games) and find evidence for the selection of payoff-dominant NE. Battalio et al. (2001) conclude that the payoff-dominant NE is more likely to be played if the optimization premium (the payoff gain from best-responding) is low. Haruvy and Stahl (2004) observe significantly more risk-dominant NE in symmetric normal-form games; however, the best predictor of subjects' behavior was an inductive dynamic learning rule. Table 7 presents the probability of a risk-dominant equilibrium (vs. a payoff-dominant equilibrium) conditional on the training set.

Result 9: Prior experience with coordination games, such as SH, CH, BOS, or games without conflict, such as NC, significantly increases the likelihood of playing payoff-dominant equilibria in stag-hunt games.

**Table 7:**
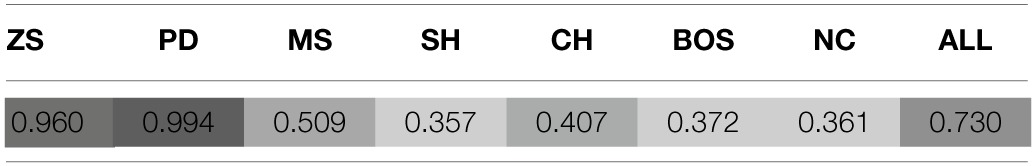
**Probability of risk- vs. payoff-dominant equilibria in SH games**.

The higher a cell's value, the darker the shading.

Games with conflicting incentives, by their nature, emphasize the risk of an opponent unilaterally deviating from a socially optimal outcome in the pursuit of self-interest—the prime example is the prisoner's dilemma game. Bednar et al. (2012) present experimental evidence that Pareto efficient outcomes are more likely to be achieved when prior experience is with the NC class (they refer to these games as self-interest games). This result is corroborated in the context of equilibrium selection as the highest probability of achieving the PDNE (outside of training on SH games) occurs when the NNs are trained on the NC game class.

Result 10^*^: Prior experience with game classes involving conflict, such as ZS and PD, significantly increases the likelihood of playing risk-dominant equilibria in stag-hunt games.

The probability of playing the RDNE is essentially one (barring minimal errors from the stochastic specification) for the ZS and PD classes. Training the NN agents on ALL game classes also leads to a strong preference for risk-dominant equilibria (0.73).

### 4.7. Deep vs. superficial transfer of learning

Deep learning may be defined in various ways, in this section I will focus on a simple, strict definition. Deep ToL is said to occur if agents learn to play the unique PSNE in the training set and continue to do so with similar probability when exposed to a new game class with a unique PSNE.

Result 11^*^: No across-class deep transfer of learning (as defined by transfer of PSNE behavior) is observed between the PD and NC game classes.

There exist two game classes that have exactly one PSNE, PD and NC. Table 8 replicates the comparisons of the joint probability of playing the PSNE from Table 5 for ease of comparison. Significant transfer of learning requires that the numbers within each row (training class) of the table be very similar. This is not the case across PD and NC classes as testing on a different game class led to a virtually zero probability of playing the PSNE. This is a strong result indicating no deep ToL *across* these game classes.

Result 12^*^: Within-class transfer of learning is observed for the PD and NC game classes.

**Table 8:** **Transfer of learning in games with a unique PSNE (probability of joint PSNE play)**.

**Class**	**Testing**	**PD**	**NC**	**ALL**
Training	PD	0.998	0.004	0.532
	NC	0.008	0.999	0.413
	ALL	0.901	0.844	0.83

Agents trained and tested on the same class consistently played the PSNE (the leading diagonal in Table 8). Consequently, agents appear to be exhibiting deep ToL *within* these two game classes. On the surface, Results 11 and 12 may appear perplexing—if agents exhibit deep within-class ToL why does this not translate to deep across-class ToL? A hypothesis that resolves this is that training on a single game class does not afford the NN agents an opportunity to truly learn to solve for the PSNE, but rather they may have found a simple heuristic that happens to coincide with the PSNE for that particular game class. However, the predictions of the heuristic and the PSNE may diverge for other game classes. For example, choosing the social optimum in the NC game class perfectly coincides with the PSNE, but the social optimum in the PD game does not prescribe the PSNE solution. Results 11^*^ and 12^*^ are consistent with a hypothesis that simultaneous exposure to a variety of game classes is a necessary condition for across-class deep ToL. This hypothesis is further supported in the next result.

Result 13^*^: Significant across-class deep ToL occurs if NNs are trained on the ALL set and subsequently play either the PD or NC game class. Deep ToL is inhibited by an impoverished or highly strategically homogenous set of inputs (i.e., training games).

I test this hypothesis by examining the behavior of NNs trained on the ALL set when presented with the test sets ALL, PD, and NC. Firstly, note that training and testing on the ALL set leads to a high probability of playing the PSNE, 0.83. In contrast to Result 11^*^, NN agents trained on ALL showed a similarly high probability of playing the PSNE for *both* the PD and NC classes, 0.901 and 0.844 respectively. I conclude that NNs are capable of deep ToL if their training game set is rich enough, as measured by the diversity of games with significantly different strategic characteristics. Conversely, an impoverished set of stimuli is not conducive to learning deep concepts such as the Nash equilibrium. Spiliopoulos (2011b) also finds indirect evidence supportive of this claim for NNs trained on 3 × 3 games drawn randomly from any game class. The behavior of the trained NNs was conditional on the number and type of PSNE implying that the NNs had endogenously learned the different strategic characteristics of game classes.

I now examine the relationship between superficial and deep transfer of learning for a wider array of games. In the context of these simulations, learning is defined as superficial if NN agents continue playing an action with similar probability, despite a change in the strategic characteristics of a game. Deep transfer of learning manifests as choice probabilities that are strongly conditional on the game class, even if games exhibit superficial payoff-similarity. I use a sequence of games derived from simple transformations of different classes of games into each other (Bruns, 2015) to investigate the type and degree of ToL. I chose four transformations forming a closed loop, in the sense that after all the transformations are performed the initial game is reproduced. This loop contains four different game classes: PD, SH, NC and CH. The games were chosen on the following basis: (a) to minimize the number of payoffs that must be changed for the transformation, (b) to ensure that payoffs are symmetric about zero and that their range is not near the maximum values of −1 and 1, where neural saturation may diminish the responsiveness to payoff changes. Each of the transformations in Table 9 requires changing only four of the payoff outcomes in a game and each transformation induces a maximum change in the rank of coupled payoffs of value one, i.e., the best outcome may be transformed to the second-best outcome, the second-best outcome to the first- or third-best only, and so forth. Games were transformed by incrementing the necessary payoffs by the following increments λ = {0.01, 0.02, …, 0.23, 0.24}. Also, note that the maximum difference for the payoffs in any cell for any pairing of games is 0.5, which is only 25% of the permissible input range of values.

**Table 9:**
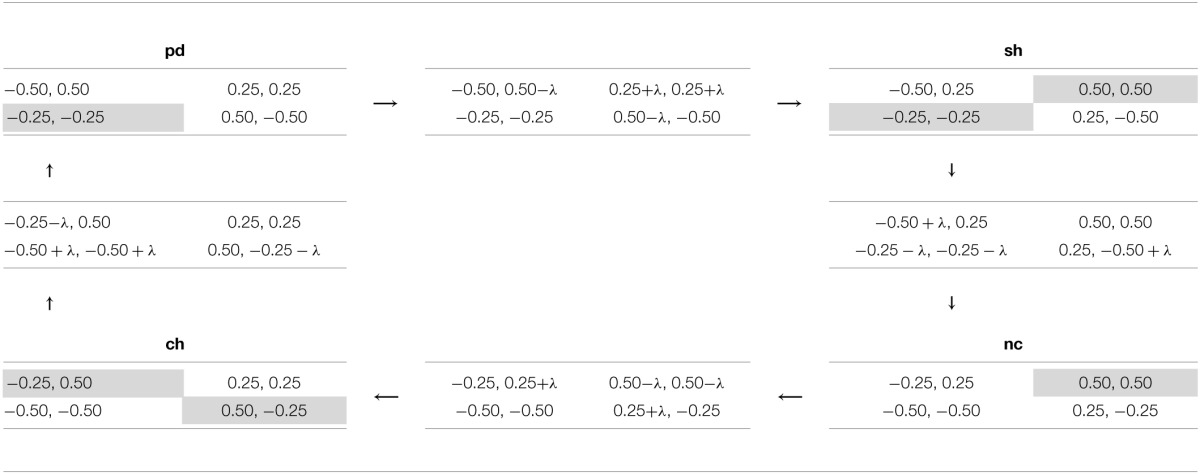
**A sequence of games spanning four game classes**.

Shaded cells correspond to pure-strategy Nash equilibria of the games λ = {0.01, 0.02, …, 0.23, 0.24}.

The higher a cell's value, the darker the shading.

**Figure 4:**
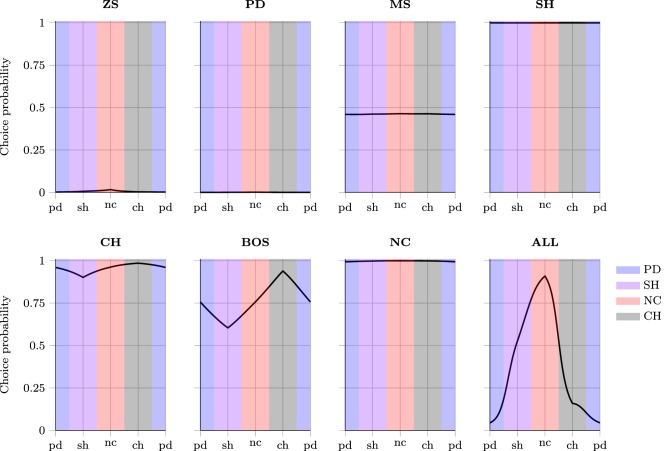
**Deep vs. superficial transfer of learning in a sequence of games**.

In Figure 4, each subgraph corresponds to agents trained on one particular set of training data. The y-axis denotes the probability of the NNs playing the first action as the row player in the games presented in Table 9. The x-axis denotes the games by increasing λ from left to right as they are transformed starting from pd to sh to nc to ch and finishing again at pd (as in Table 9). During the process there comes a point where one game class is transformed into another—in Figure 4 this occurs at the border where the shading changes color. Note, for all games at equidistant points along the x-axis on either side of this boundary, the differences in payoffs with the game on the boundary are the same, i.e., games should be considered as similar according to a superficial payoff metric. Therefore, different choice probabilities for any such games (at equal distance from the boundary) imply that NNs must have learned that the games at these two points have different strategic characteristics, signifying deep transfer of learning rather than superficial similarity-based ToL.

Result 14^*^: Agents trained on one of the following game classes (ZS, PD, MS, SH, CH, NC) exhibited only superficial transfer of learning across game classes.

A relatively flat profile in Figure 4 indicates that despite significant changes in the strategic characteristics of the games, the agents continued behaving similarly. This suggests that agents are “action-bundling” both within and across game classes^11^. Consequently, agents trained on these game classes have not learned to distinguish and adapt their behavior to the underlying strategic characteristics of games, but rather relied upon payoff-similarity or superficial ToL. As hypothesized above, this is likely driven by the information-poor learning environment that results from exposing agents only to a single game class.

Result 15^*^: Deep across-class transfer of learning, driven by the emergent notion of the strategic characteristics of different game classes, is most clearly found when agents are trained on the ALL set of games, and less so for BOS games.

The ALL training class exhibited the largest variation in choice probabilities in Figure 4 and agents' behavior was clearly conditional on the game class they were tested on. Also, NNs had significantly different choice probabilities for superficially similar games (equidistant from the boundaries where game classes changed). Both of these observations are consistent with deep ToL. However, if deep ToL were the only mechanism in operation, there should be an abrupt rather than smooth change in choice probabilities at the marginal game straddling two adjacent game classes. The smoothness in the behavioral change points to a combination of deep *and* superficial ToL in operation. Therefore, deep and superficial ToL should not be viewed as mutually exclusive, or as capturing some fundamental dichotomy; both may be in operation simultaneously and experiential learning may lead to gradual transitions from one to the other.

## 5. Discussion

This paper presented evidence that a parallel-distributed learning model of agents playing 2 × 2 normal form games accounted for many of the existing experimental findings regarding transfer of learning from previously seen games to new games with different strategic characteristics. Specifically, the robust empirical finding that cooperation in games where it is not the Nash equilibrium, e.g., Prisoner's dilemma, is more likely when players have prior experience with coordination games was corroborated (Knez and Camerer, 2000; Ahn et al., 2001; Devetag, 2005; Bednar et al., 2012; Cason et al., 2012; Cason and Gangadharan, 2013; Juvina et al., 2013). Simulating agents' behavior allowed for more specific predictions regarding the effects of transfer of learning for seven specific classes of games. Prior exposure to zero-sum, Prisoner's dilemma and discoordination games negatively impacted the level of cooperation (and realized payoffs) in *all* the types of games. Conversely, experience with games that promoted cooperation rather than conflict encouraged higher levels of cooperation (and in most cases higher payoffs) in new game classes even when the new incentive structure was competitive. Furthermore, the model predicted that equilibrium selection in Stag-Hunt games is also experience-dependent. Prior exposure to zero-sum and Prisoner's Dilemma games led to a higher probability of actions associated with the risk-dominant rather than payoff-dominant Nash equilibrium. This is a novel prediction that can be investigated in future experimental work with real subjects.

The connectionist literature has debated the benefits of “starting small”, either in terms of an initially constrained network architecture or exposure to an easier training set (Elman, 1993; Rohde and Plaut, 1999). I find that “starting big” in terms of a diverse set of games was a necessary condition for deep learning of the strategic implications of different games, playing the appropriate response (Nash equilibrium) and transferring this behavior to new games. In earlier work, “starting big” in terms of network architecture was also found to be a necessary condition for deep learning (Spiliopoulos, 2011b, 2012). These studies found that networks with fewer hidden layers and fewer neurons per layer (e.g., at the extreme, perceptrons with no hidden layer) were significantly more likely to play a dominated action and not play a Nash equilibrium of the game.

Deep learning of representations and concepts is usually implicitly associated with better outcomes in existing applications in the literature, such as the acquisition of language. This relationship does not hold for strategic interactions with other agents since outcomes depend on the collective actions of all players of the game. Promoting deep learning in the agents (through exposure to a strategically diverse set of games) led to a higher rate of Nash equilibrium behavior, which in many game classes is detrimental, e.g., Prisoner's dilemma. Consequently, the encouragement of superficial rather than deep learning, for example, by training networks only on the stag-hunt game, led to better outcomes on average in other game classes.

Future research can aim at a closer alignment with developmental psychology such as the computational modeling of developmental trajectories across the lifespan. This can be accomplished by a detailed examination of the behavior of connectionist models of strategic decision making as a function of their level of experience. The effects of aging and brain disorders can also be investigated in a similar fashion to existing research for other tasks–see the discussion in Munakata and McClelland (2003)–by varying the parameters of the neural networks such as the backpropagation learning rate, the number of connected neurons and hidden layers, or neuronal sensitivity via the transfer function. Furthermore, in this paper I examined only the *initial* transfer of learning that occurs when an agent is suddenly forced to play a new class of games. An important extension would be to further simulate learning in the new class of games, and document the learning trajectory and emergent long-run behavior. Another possibility is to look at how agents with little experience fare if they are suddenly moved to a new population of agents with much more experience, and vice-versa. Further extensions could include endogenous matching of players rather than the random rematching used in this paper. This would highlight the importance of the emergence of networks of players with different rates of interaction and its effect on learned behavior. Connectionist models of decision making are also useful for modeling how preferences are constructed, or arise endogenously, as a function of the environment, e.g., the types of decisions or problems they are facing and how other agents are behaving. Simulations with a systematic manipulation of key properties of the environment and agents could also shed light on the coupling of the two in the spirit of procedural rationality (Simon, 1976, 1986).

### Conflict of interest statement

The author declares that the research was conducted in the absence of any commercial or financial relationships that could be construed as a potential conflict of interest.

## Figures and Tables

**Figure A1:**
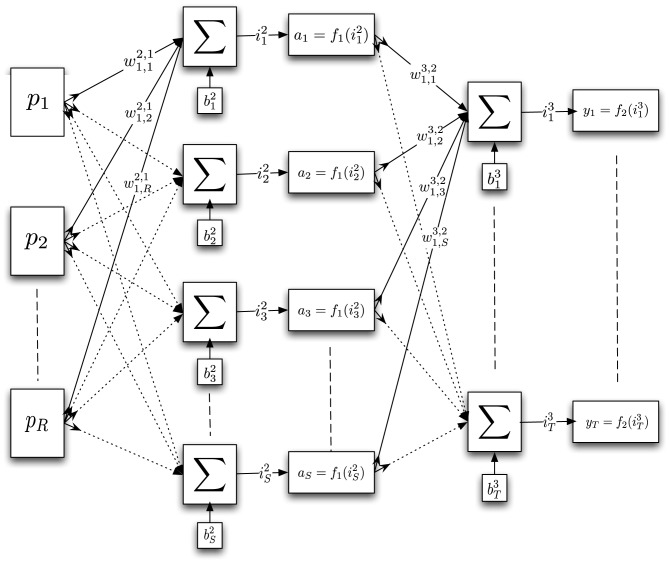
**Detailed structure and topology of feedforward neural networks**.

## Footnotes

^1^I refer to the resurgence of connectionist research after the so-called AI-winter, which is widely considered to have been triggered by the discussion in Minsky and Papert (1969) of the limitations of perceptrons and the training algorithms known at the time.

^2^Of course, simulations–as any other methodology–have their own limitations. Consequently, the three approaches are complementary rather than antagonistic, and a synthesis of these is valuable.

^3^Izquierdo et al. (2012) refer to this as Learning Game Theory. Another approach, Evolutionary Game Theory, assumes learning is driven by external evolutionary forces. Learning Game Theory is more appropriate for ToL as it is inextricably linked both to the types of games DMs are exposed to and their opponents' behavior in said games.

^4^For example, learning to perform iterated deletion of dominated strategies in one game will not be useful in another game that is not dominance-solvable.

^5^Early experimental work in the field focused on the most natural, and easiest to examine, environment where learning is expected to be important, e.g., a repeated game with fixed partner matching. The use of an unchanging stage game allows for a relatively easy formulation of learning models. For example, both belief formation and reinforcement learning algorithms require unchanging action and payoff structures. If the payoffs of the game or available actions were suddenly different these models could not condition learning upon these changes. However, this simplicity comes at the cost of examining a very narrow subset of possible games and learning situations.

^6^Another related argument in favor of algorithms that may not accurately reflect biological mechanisms is that despite this the neural networks derived from backpropagation may be a relatively accurate reflection of the networks that arise in the the human mind (Lehky and Sejnowski, 1988; Kettner et al., 1993).

^7^Generic bimatrix games have an odd number of NE, whereas non-generic games may have an even number. Our method of sampling games guarantees that they are generic.

^8^For example, Zizzo (2003) argues that empirical evidence supports the hypothesis that social preferences are determined to a large extent by the environment.

^9^Since the error is zero, substituting (*y*_*t*_ − *z*_*t*_) = 0 into Equations A8 and A12 implies that the weight adjustment given by Equation (A4) is also zero.

^10^Due to the stochastic output of the NNs, the correlation coefficient will not necessarily be equal to one.

^11^Note, the original definition of action-bundling applied to within game class behavior (Grimm and Mengel, 2012).

## Appendix A

### An introduction to neural networks

Figure A1 displays the topology of the feedforward neural networks used. The leftmost layer in the diagram is the input layer and it consists of input neurons denoted by *p*_*r*_ where *r* = 1, …, *R*. Each *p*_*r*_ is the payoff for a specific player from a specific cell of a game; hence, *R* = 8 for the 2 × 2 games examined in this paper.

The second layer–a hidden layer–consists of *S* neurons maximally connected to the input neurons. There is a total of *R* · *S* connections between the first and second layers. Each connection is associated with a weight, *w*^2, 1^_*s*, *r*_; the subscript *s*, *r* refers to a connection from the *rth* neuron to the *sth* neuron and the superscript 2, 1 indicates that these weights connect the first and second layers of the NN. The activation of each neuron in the second layer, *i*^2^_*s*_, is the summation of the product of the inputs and their corresponding weights plus a constant or bias, *b*^2^_*s*_. Hence, for each of *S* neurons in the second or hidden layer:

(A1) $$i_{s}^{2}=b_{s}^{2}+\sum_{r\;=\;1}^{R}w_{r,s}^{2,1}\;\cdot \;p_{r}$$

Inputs are passed through a hyperbolic tangent sigmoid (or tansig) function, *f*_1_(*i*_*s*_) = 2 · (1 + *e*^− 2*i*^2^_*s*_^)^−1^ − 1, that maps the domain (−∞, + ∞) to the range (−1, 1). The outputs, *a*_*s*_, are passed to the *T* neurons in the final or output layer. Each neuron outputs the probability of choosing any action, i.e., *T* = 2 for the games in this study. Neurons in the output layer are connected to every neuron in the second layer with connection weights, *w*^3, 2^_*s*, *t*_. The input to each *t* neuron is the summation of product of the outputs, *a*_*s*_, and the corresponding weights, *w*^3, 2^_*s*, *t*_ plus a bias *b*^3^_*t*_:

(A1) $$i_{t}^{3}=b_{t}^{3}+\sum_{s\;=\;1}^{S}w_{s,t}^{3,2}\;\cdot \;a_{s}$$

These inputs are transformed by the function *f*_2_ into the final outputs of the NN, *y*_*t*_. Since the outputs correspond to a probability distribution over the action space, *f*_2_ is chosen to be a softmax function where $y_{t}=f_{2}(i_{t}^{3})=\frac{e^{i_{t}^{3}}}{\sum_{t\;=\;1}^{T}e^{i_{t}^{3}}}$ so that $\sum_{t\;=\;1}^{T}y_{t}=1$.

For ease of exposition, the network presented above had only one hidden layer. However, the NNs in this study consist of three hidden layers, each with the same structural and functional properties, to increase their ability to approximate arbitrarily complex functions.

Information flows forwards through the neural network (from left to right in the diagram) but the backpropagation rule sends error signals backwards through the network. After making a choice the backpropagation algorithm compares the values of the neurons at the output layer to the desired values and adjusts the connection weights to reduce the error of the NN. The backpropagation learning rule uses the chain rule to assign the contribution of each neuron to the observed error, thereby calculating how connection weights should be changed to decrease the error. A gradient descent method is used to modify weights so that the network successively approaches a state where the error function attains a minimum. Note, that this algorithm is not immune to the possibility of settling on local rather than global minima.

## Appendix B

### The backpropagation algorithm

NNs store distributed–not localized–knowledge in the weights and biases of the neurons that are updated via supervised learning after the presentation of each set of inputs, i.e., the game's payoff matrix. For each set of inputs, *P* = {*p*_1_, …, *p*_*R*_}, there exists a set of ideal outputs, *Z* = {*z*_1_, …, *z*_*T*_}. Selten (1998) argues that a general principle guiding learning is ex-post rationality, where an agent adjusts his/her behavior in the direction of the ex-post best response to the immediately prior outcome. Learning direction theory has been used to explain the Winner's Curse (Selten et al., 2005), to model how agents learn to allocate resources (Rieskamp et al., 2003), to model behavior in guessing games (Nagel, 1995) and auctions (Ockenfels and Selten, 2005).

Ex-post rationality suggests that the ideal output is the hypothetical best response of the NN given its opponent's last action. Therefore, for each game exactly one *z*_*t*_ is equal to one and the rest are zero. Define the mean square error, *E*, of the network, to be:

(A3) $$E=\frac{1}{2}\;\cdot \;\sum_{t\;=\;1}^{T}{\left(z_{t}-y_{t}\right)}^{2}$$

The backpropagation algorithm uses gradient descent to adjust weights according to the following equation:

(A4) $$△w_{\cdot ,\cdot }^{\cdot ,\cdot }=-\eta \frac{\partial E}{\partial w_{\cdot ,\cdot }^{\cdot ,\cdot }}$$

The weight adjustment depends on the negative of the gradient of the error function and on its magnitude,. The step size (or learning rate) η is a constant controlling the magnitude of the adjustment. Hence, weights are updated in the direction which reduces the error, *E*, and the magnitude of the change is dependent on the sensitivity of the error function to small changes in the weight. The necessary algebra to derive ∂*E*/∂*w* for both output layer and hidden layer neurons is presented below.

The chain rule leads to the following derivation for output layer weights:

(A5) $$\frac{\partial E}{\partial w_{s,t}^{3,2}}=\frac{\partial E}{\partial y_{t}}\frac{\partial y_{t}}{\partial i_{t}^{3}}\frac{\partial i_{t}^{3}}{\partial w_{s,t}^{3,2}}$$

However, from Equation (A2):

(A6) $$\frac{\partial i_{t}^{3}}{\partial w_{s,t}^{3,2}}=a_{s}$$

and from Equation (A3):

(A7) $$\frac{\partial E}{\partial y_{t}}=(y_{t}-z_{t})$$

Substituting these Equations into Equation (A5) results in:

(A8) $$\frac{\partial E}{\partial w_{s,t}^{3,2}}=(y_{t}-z_{t})a_{s}f_{2}^{\prime }(i_{t}^{3})$$

Calculations for weights in hidden layers are more involved as the desired output of such neurons needs to be calculated. The analog of Equation (A5) for a hidden layer neuron is:

(A9) $$\frac{\partial E}{\partial w_{s,r}^{2,1}}=\sum_{t=1}^{T}\frac{\partial E}{\partial y_{t}}\frac{\partial y_{t}}{\partial a_{s}}\frac{\partial a_{s}}{\partial w_{s,r}^{2,1}}$$

This equation requires a summation of terms over *t* due to the propagation of the effect of *w*^2, 1^_*s*, *r*_ through the interconnections between the *sth* neuron and all *T* neurons in the output layer. The derivative of the output of the *sth* neuron w.r.t each weight is:

(A10) $$\frac{\partial a_{s}^{2}}{\partial w_{s,r}^{2,1}}=p_{r}f_{1}^{\prime }(i_{s}^{2})$$

The derivative of the error function w.r.t. the output of final layer neurons, ∂*E*/∂*y*_*t*_, is given by Equation (A7). The derivative of the output of each final layer neuron w.r.t. hidden layer neurons' output is:

(A11) $$\frac{\partial y_{t}}{\partial w_{s,r}^{2,1}}=w_{t,s}^{3,2}f_{2}^{\prime }(i_{t}^{3})$$

Finally, substituting Equations (A10, A7) and (A11) into Equation (A9) results in the following equation, which is well defined for the differentiable functions *f*_1_ and *f*_2_:

(A12) $$\frac{\partial E}{\partial w_{s,r}^{2,1}}=p_{r}f_{1}^{\prime }(i_{t}^{2})\sum_{t=1}^{T}\left(y_{t}-z_{t}\right)w_{t,s}^{3,2}f_{2}^{\prime }(i_{t}^{3})$$
